# Carbon Sphere Template Derived Hollow Nanostructure for Photocatalysis and Gas Sensing

**DOI:** 10.3390/nano10020378

**Published:** 2020-02-21

**Authors:** Zirui Lou, Yichen Wang, Yingchen Yang, Yanwen Wang, Chao Qin, Rong Liang, Xuehua Chen, Zhizhen Ye, Liping Zhu

**Affiliations:** State Key Laboratory of Silicon Materials, School of Materials Science & Engineering, Zhejiang University, Hangzhou 310027, China; zrlou@zju.edu.cn (Z.L.); pifuwuze@zju.edu.cn (Y.W.); ycyang@zju.edu.cn (Y.Y.); 21826067@zju.edu.cn (Y.W.); 21626044@zju.edu.cn (C.Q.); liangr@zju.edu.cn (R.L.); threed_cxh@zju.edu.cn (X.C.); yezz@zju.edu.cn (Z.Y.)

**Keywords:** carbon sphere, hollow structure, doping, solid solution, heterostructure, surface modification

## Abstract

As a green and preferred technology for energy crisis and environmental issues, continuous research on photocatalysis and gas sensing has come forth at an explosive rate. Thus far, promising synthetic methods have enabled various designs and preparations of semiconductor-based nanostructure which have shown superior activity. This review summarized various synthetic routines toward carbon sphere template derived hollow nanostructures and their successful attempts in synthesize doping, solid solution, heterostructure, and surface modified nanostructures for heterogeneous photocatalysis and gas sensing. Moreover, the challenges and future prospects are briefly discussed. It is eagerly anticipated that this review may broaden the view and in-depth understanding of carbon sphere template derived hollow nanostructures while expected to have further progresses in heterogeneous photocatalysis, gas sensing and other related fields which will make great contributions to their application.

## 1. Introduction

Developing sustainable energy is one of the most urgent tasks of humankinds. With the development of science and technology, the explosive growth of energy demand is making limited traditional fossil energy stretched thin. Among various types of sustainable energy, solar energy is the most abundant renewable energy source on earth. However, it is dilute, unequally distributed, and intermittent. Hence, to harness solar energy facilely, efficiently and economically remains a challenge [1]. Solar driven photocatalysis based on particulate photocatalysts is a potentially scale-up industrial and economically practical technology to convert solar energy into chemical energy. Since 1972, when Fujishima and Honda first reported the phenomenon of hydrogen generation from the decomposition of water by TiO_2_ photocatalysts, photocatalysis has gradually attracted the attention of academia and industry [2]. In 2001, Zou et al. [3] first discovered In_1-*x*_Ni*_x_*TaO_4_ photocatalyst with visible light activity for photolytic water splitting. The past two decades, various kinds of semiconductor photocatalysts have been widely studied, such as oxides [4,5,6,7,8,9], sulfides [10,11,12,13,14], nitrides [15,16,17,18], carbides [15,19,20,21], etc. Domen’s recent studies based on particulate photocatalyst sheets provide a possible way for the large-scale and practical photocatalytic water splitting [22,23,24].

A typical photocatalytic process mainly consists of three steps: (i) semiconductors absorb light of corresponding wavelength with appropriate bandgap; (ii) transition of photo induced carriers from valence band to conduction band; (iii) carrier migration to material surface for redox reaction with reactants [25]. However, the band gaps of most metal oxides are too large to avail adequate absorption of the majority of photons in the solar spectrum [26]. There are limitations in most semiconductor photocatalysts like fast charge carriers recombination, surface carrier recombination, as well as surface state pinning which prevent photogenerated carriers from participating in redox reaction [25]. In the study of particle photocatalysts, however, photocatalytic quantum efficiency of the bulk photocatalysts is limited by carrier migration, trapping, and recombination inside the bulks which account for most of the volume [27]. The nano form photocatalysts, with dimensions reaching tens of nanometers in scale, or even less than their corresponding Bohr radii, ought to possess enhanced photocatalytic quantum efficiency [28]. On account of the higher specific surface area and the smaller grain size, more surface catalytic sites and shorter distances from interior to surface of the material enable photo induced carriers to participate in surface redox reaction instead of loss in bulks. Similarly, designing and constructing nanostructures is the trend of enhancing sensing performance of semiconductor-based gas sensors. 

To this end, researchers have synthesized nanomaterials by various methods and improved the efficiency of solar absorption and conversion efficiency of the photocatalysts by doping, constructing heterostructures and surface modification. Among them, carbon sphere template derived hollow nanostructure is one of the most promising methods to prepare high-efficiency photocatalysts and gas sensors due to some unique characteristics. First of all, the raw materials (any of the saccharides and some biomass) and products of template (carbon sphere) are environmentally friendly. Then, the surface functional groups adsorb metal ions uniformly and non-selectively, so we can realize doping, solid-solution, heterostructure, and surface modification easily by simply adjust the adsorption or annealing conditions. In view of these facts, we introduce a synthetic method that synthesizes a variety of multiple metal compound hollow spheres by using ions adsorption and templating approaches. This strategy could avoid the bulk form of the multiple metal compound in traditional synthesis process and tune the composition of different metals precisely. These multiple metal compound hollow spheres have shells in nanoscale and high specific surface areas, which bound to be helpful for photocatalysis, gas sensing, and other related reactions.

In this review, we will first give an overview on the synthesis of carbon sphere template and then summarized various synthetic routines toward carbon sphere template derived hollow nanostructures, including their advantages in synthesizing different types of nanostructure for photocatalysis (doping, solid-solution, heterostructure, surface modification). Secondarily, their valuable applications in gas sensing using the same idea as photocatalyst modification will be summarized and discussed. At the end of this review, challenges and outlooks in the field of carbon sphere template derived hollow nanostructure are highlighted.

## 2. Carbon Sphere Template—Overview of Synthesis

Carbon spheres have been widely researched and applied to various fields—including batteries, photocatalysis, capacitors, and fuel cells—due to their easily available sources and facile synthesis. The last few decades have witnessed a rapid growth in sustainable energy field and this has led to the research of various application and approaches of carbon spheres, including carbonization routes [29], chemical vapor deposition (CVD) [30], ultrasonic-spray [31], and hydrothermal carbonization (HTC) [6,32,33]. Among these methods, hydrothermal carbonization is the one that aroused the greatest interest due to its facile procedure and mild experimental condition. Besides, carbon spheres obtained through HTC method have tunable diameters, great monodispersity, and excellent surface activities compared to carbon spheres synthesized by other procedures. Accordingly, HTC method is considered a perfect way to obtain carbon spheres as templates and assist the subsequent modification.

**Figure 1:**
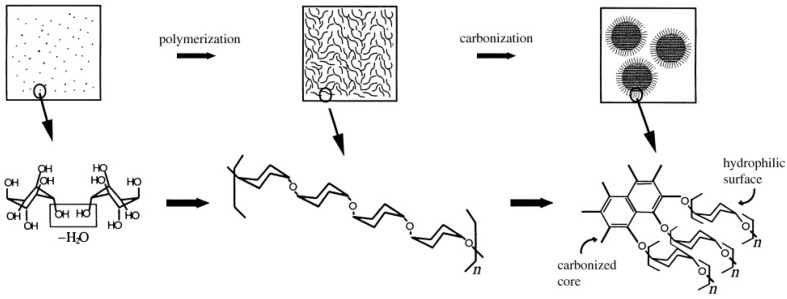
Schematics of the possible synthesis procedure of carbon spheres. Reproduced with permission from [32]. Copyright WILEY, 2004.

Wang et al. [34] successfully synthesized a novel monodispersed hard carbon spheres which is perfectly round with good surface condition and evenly distributed nanopores. In this experiment, carbon spheres were facilely prepared by two steps, including dehydration at low temperatures and carbonization at high temperatures of glucose. Sun et al. [32] put forward a simplified hydrothermal procedure to synthesize micro- or nanospheres. Carbon nano- and microspheres were synthesized directly using aqueous glucose solution as precursor by the method of hydrothermal synthesis at 160–180 °C. The growth model of carbon spheres is shown schematically in Figure 1. The synthesis process is facile, and the as-prepared hard carbon spheres were tunable in diameters, crystallinity, chemical composition, with reactive surface and inherited functional groups of the precursor. Titirici et al. [35] reported a one-pot synthesis to obtain hollow spherical metal oxides such as Fe_2_O_3_, Ni_2_O_3_, Co_3_O_4_, CeO_2_, MgO, and CuO via a hydrothermal approach by directly adding glucose or sucrose as precursor and metal oxide precursors. It is to be observed that, during the synthesis procedure, the carbon spheres were also formed and serve as templates and this method is more simplified but it has no harmful effects for metal oxide hollow spheres. Although the hydrothermal carbonization (HTC) method for carbon spheres is mature and widely used, there exist some limitations that restrict the formation of relatively small size of carbon sphere. Tan et al. [36] finally overcome this barrier by varying parameters of hydrothermal carbonization process—including glucose concentration, pH value, and temperature—and synthesize small-sized carbon spheres (40 nm) for the first time.

A typical hydrothermal synthesis for carbon sphere template consists of 5g glucose dissolving in 30 ml water, sealing in a 50 mL autoclave and maintaining at 180 ℃ for 6 h. The as-prepared templates were isolated by several rinsing-centrifugation cycles until the solution is clear and oven-dried. Among some details of the experiment, we found the dispersion of the template can be controlled by trace amount of surfactant, solubility of glucose and quenching of the autoclave.

**Figure 2:**
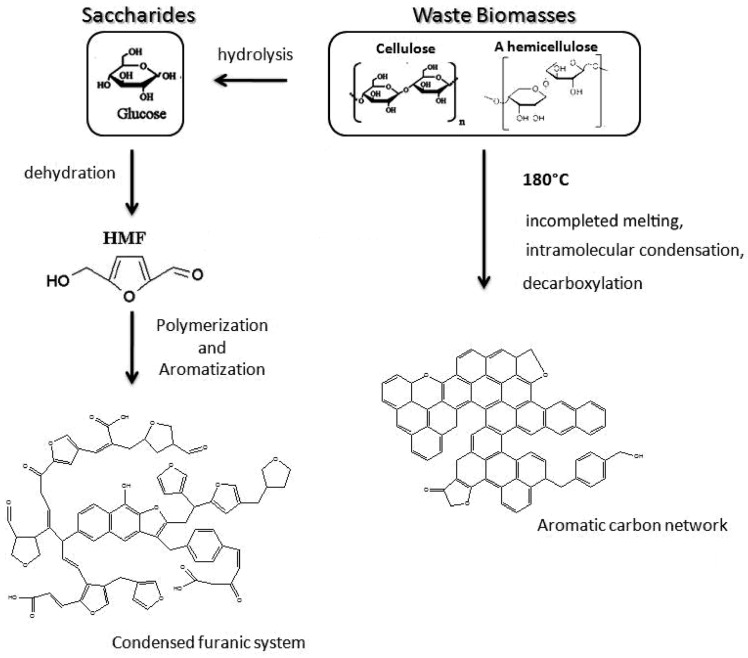
Proposed formation mechanism of sphere-like carbon synthesized from saccharides and biomasses. Reproduced with permission from [39]. Copyright American Chemical Society, 2012.

Besides, surface property of carbon spheres and precursors of carbon spheres have been widely studied in recent years. The surface of carbon spheres, especially those synthesized from hydrothermal reaction, possess high density of hydrophilic groups such as –OH and C=O groups, so that can absorb various of metal ions easily and uniformly without any further modification [37]. This is the critical superiority when carbon spheres are applied as templates. Moreover, many researchers are focusing on a wide range of source to obtain carbon spheres by hydrothermal reaction, including general monosaccharide (xylose [38], glucose [38]), disaccharides (sucrose [6,38], lactose [39]), polysaccharides (starch [40,41], cellulose [42]), and some biomasses [39]. The possible formation procedure is shown schematically in Figure 2. These studies showed that all these sources could be applied to synthesize sphere-like carbon sphere-like structures which contained amounts of hydrophilic reactive functional group on the surface, but the qualities (such as morphology and dispersivity) of carbon spheres are better when saccharides are chosen as precursors due to the structural complexity of biomasses [39]. Despite all of this, using biomasses to synthesize carbon spheres will continue to be a hot spot because of its abundant resources, environmental friendliness, and consistency of sustainable development.

In addition, there are also some reports about sphere-like carbon with some changes of morphology, including mesoporous carbon, hollow carbon spheres, which can also be applied as templates [43,44,45]. Mesoporous and hollow carbon spheres are synthesized by a numerous methods, such as carbonization and a template procedure. Mesoporous hollow carbon spheres could be obtained through carbonization of polystyrene (PS) [43]. Carbon hollow spheres with tunable diameter and surface properties have studied and prepared with the use of templates such as silica spheres, some polymer spheres, metallic templates [44], and some other core templates [45]. There is still great necessity to deeply research the preparation of various carbon spheres (both morphology and property) to realize a synthesis method that starts with environmental-friendly materials and involves a facile synthesis process.

## 3. Carbon Sphere Template Derived Hollow Nanostructure: Advantages in Synthesizing Different Types of Nanostructures for Photocatalysis

**Figure 3:**
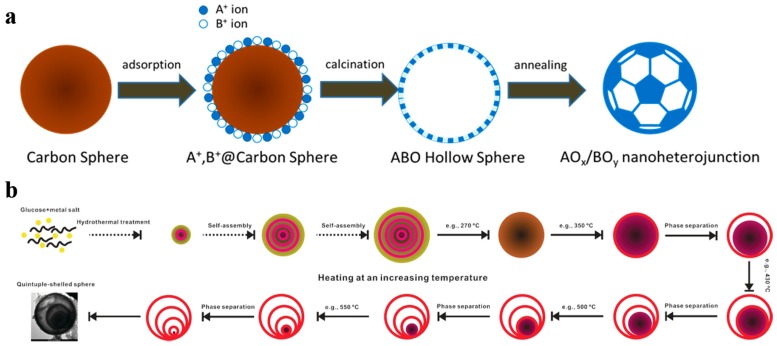
General process for the formation of carbon sphere template derived (**a**) hollow nanoheterostructure and (**b**) controllable multilayer structure. Reproduced with permission from [47]. Copyright WILEY, 2016.

From recent studies, the nanoscale morphology could conducive to enhance the charge carriers’ separation and transfer efficiency as well as raise the density of catalytic sites and it is one of the keys to improve photocatalytic performances [27,28,37]. However, for these traditional methods such as sol–gel and sintering methods, a precipitation or solid-state reaction process is essential to mix the different metal ions on molecular level. Resultantly, the large particle size and the aggregation phenomena are inevitable in multiple metal compounds [46]. Thus a general method for the synthesis of multiple metal compounds consists of doping, heterostructure, and surface modification strategies in nanoscale is essential in photocatalysis. Carbon sphere template derived hollow nanostructure is such a way to achieve the above strategies. Even controllable multi-layer hollow structures can be realized by different post-processing methods [47]. As shown in Figure 3, it is the illustration of carbon sphere template derived hollow nanoheterostructure and controllable multilayer structure.

### 3.1. Doping

Most metal oxide semiconductors with wide band gap could hardly respond to the visible light region [48], which limit their application in photocatalysis. Hence it is urgent to design semiconductor-based photocatalysts active both in UV and visible light region. Doping metal oxides (TiO_2_, ZnO, WO_3_, etc.), polymetallic oxides and nitrides [49] with non-metals (C, O, S, N, P, etc.) [48] or metals (Na, Mg, Fe, Lanthanide, etc.) [50] expand the effective absorption to visible light region to promote light harvesting and photocatalytic performance. One of many valid methods to design efficient photocatalysts which also can absorb visible light is creating impurity levels in band gap by doping [51]. We can achieve doping of many kinds of desired metals into oxides taking the characteristics of good dispersion of carbon sphere template and its uniformly and non-selectively adsorption of metal ions. By further regulating the experimental process, the doping of non-metallic elements can also be realized.

**Figure 4:**
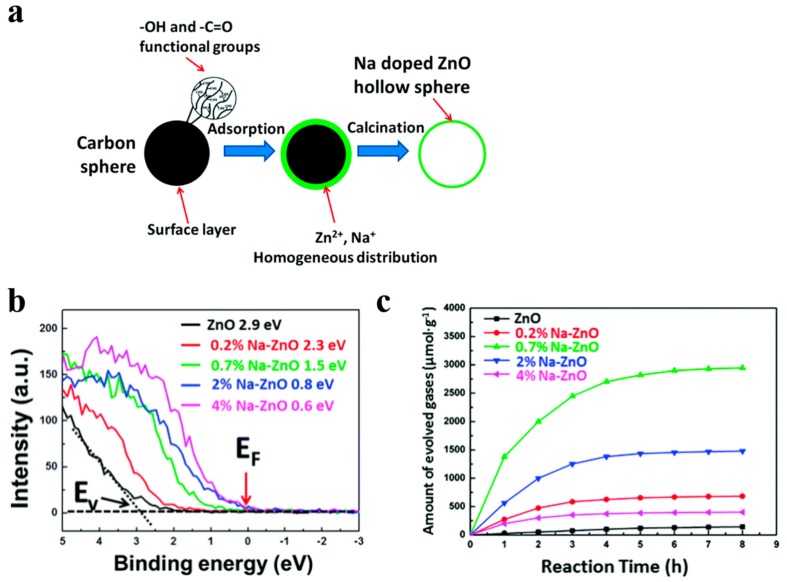
(**a**) Schematic route for preparing Na-ZnO hollow nanostructures, (**b**) valence band measure of Na-ZnO with various Na doping concentrations, (**c**) corrensponding H_2_ generation for Na-ZnO/Pt hollow spheres with 0, 0.2, 0.7, 2, and 4 at % Na. Reproduced with permission from [53]. Copyright Royal Society of Chemistry, 2016.

For instance, Song et al. [52] demonstrated that Fe doped contents can be precisely regulated by using this simple templating method, so that they acquired broader light absorption region. Fe doping introduced impurity levels in the forbidden band confirmed by DFT (density functional theory) calculation, which narrowed the band gap of WO_3_. Meanwhile, WO_3_ nanostructured photocatalysts with 5 at % Fe showed the best photocatalytic performance due to the low recombination and high transformation of the photo-generated carriers. Besides, Na-doped ZnO nanostructures have been firstly fabricated by ion adsorption and template method utilizing carbon spheres as templates [53]. Na and Zn elements cannot be precipitated simultaneously because their chemical activities in solution differ from each other. Instead of co-precipitation method, they gather the Zn and Na cations uniformly and simultaneously on the surface of carbon spheres (Figure 4a). Engineering the Zn-doped contents can tune the work function of Na-doped ZnO from 4.48 eV to 6.19 eV, and fermi level get closer to the level of valence band by the increasing of Na doping concentration, which presented the success of a *p*-type doping effect (Figure 4b). They have got Na-ZnO/Pt with 0.7 at % which presented the highest photocatalytic H_2_ generation rate and it was obviously superior to pure ZnO (Figure 4c). 

Rare earth metals also play a vital role in metal doped semiconductor-based photocatalysts. According to Chao et al. [54], Nd^3+^ led to defects in the lattice which were acted as electron traps, and also could band with non-bridging O^2−^ around the network structure. Nd^3+^ doping could make a red shift of absorption spectrum for TiO_2_. In another study [55], they used cerium instead of neodymium to dope into titania hollow spheres. Ce^4+^ ions could trap the excited electrons and then electrons would be transferred to adsorbed oxygen on the surface like the reaction

Ce^4+^+e^−^→Ce^3+^ (1)

Ce^3+^+O_2_→Ce^4+^+ •O_2_^−^ (2)

With the increase of Ce^4+^ doping content, the space charge layer would be thinner, which efficiently separates electron–hole pairs.

**Figure 5:**
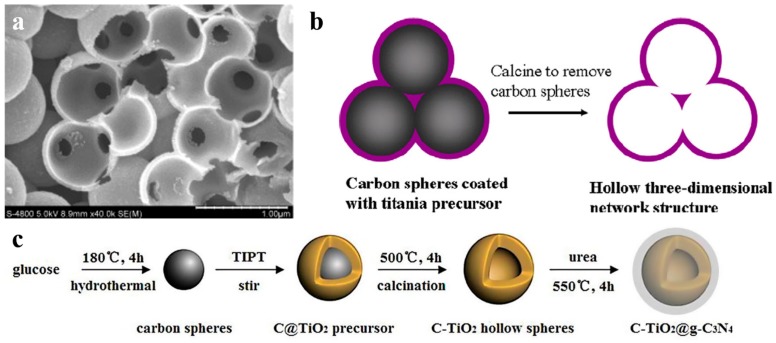
(**a**) SEM (scan electron microscope) image of broken TiO_2_ nanospheres with a macroporous channel structure by crushing THS. Reproduced with permission from [56]. Copyright WILEY, 2012. (**b**) Formation mechanism of the porous channels in the hollow 3D network structure. Reproduced with permission from [57]. Copyright Elsevier, 2012. (**c**) Schematic route for preparing C-TiO_2_@g-C_3_N_4_. Reproduced with permission from [58]. Copyright Elsevier, 2017.

Among all the non-metals used as dopant materials by using carbon spheres templating, carbon doping is relatively common. Carbon spheres are used as hard template and also provide a source of carbon doping. Shi et al. reported C-doped titania nanostructures and its photocatalytic application in 2012 [56]. They presented the preparation of carbon doped TiO_2_ nanostructures with a hierarchical macroporous channel structure (Figure 5a), which showed high potocatalytic degradation performance. In another work [57], they deduced the formation mechanism of hollow 3D network structure (Figure 5b), because the surface of carbon spheres was hydrophilic. Carbon doping narrowed the band gap of titania, and hollow 3D structure offered a pathway for guest molecules and enhanced the light harvesting. Recently, Shi’s group [58] realized C_3_N_4_ decorated carbon-doped TiO_2_ by in situ growth (Figure 5c). C doping influenced the energy band structure of TiO_2_ to enhance photocatalytic activity for application in hydrogen generation under simulated sunlight. Except carbon doping, there are a few works on non-metal doped semicondutor-based photocatalysis in hollow nanostructure, such as Br-doped TiO_2_ [59].

**Figure 6:**
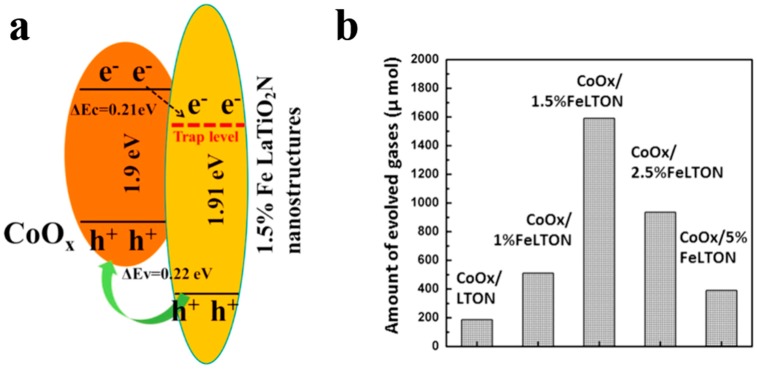
(**a**) Band energy diagram of CoO*_x_*/LaTiO_2_N with1.5% Fe, and (**b**) oxygen generation rates for samples with various Fe doping concentration under visible light illumination. Reproduced with permission from [60]. Copyright Elsevier, 2016.

**Figure 7:**
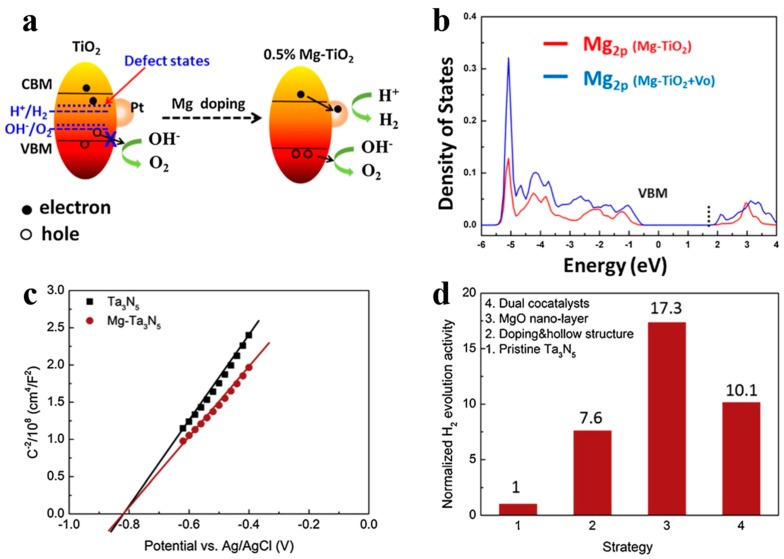
(**a**) Schematic illustration of the band structure of anatase TiO_2_ with and without Mg doping for water splitting. (**b**) The density of states of Mg 2p in different samples. Reproduced with permission from [61]. Copyright Elsevier, 2017. (**c**) Mott–Schottky plot of Ta_3_N_5_ with and without Mg dopant in dark, and (**d**) normalization of the contribution of individual strategy to the photocatalytic activity. Reproduced with permission from [62]. Copyright Elsevier, 2019.

In addition to being visible-light absorption centers, dopant also could reduce recombination losses in photocatalyst by trapping photo-generated carriers. Reciprocally, it enhanced the carriers separation to obtain the superior photocatalytic performance. The non-selectively adsorption of carbon sphere template provides a good condition for various doping. For instance, Li et al. [60] described that Fe-doped LaTiO_2_N nanostructures exhibited high efficiency, because doping can match the level of conduction band between cocatalysts and LaTiO_2_N hollow nanostructures by creating empty mid-gap states in Fe doped LaTiO_2_N. As shown in Figure 6a, Fe dopants created a defect level lower than the level of conduction band both of LaTiO_2_N and CoO*_x_*, so that the photo-generated electrons could be gathered to CoO*_x_* nanoparticles. As a result, the sample with 1.5% Fe doping exhibited quantum efficiency as high as 55% for O_2_ evolution which was fairly higher than other LaTiO_2_N system (Figure 6b). Besides, dopant also can reduce the defect sites, where are electron–hole recombination’s sites. Gao et al. [61] found that Mg dopants could not create new trapping states in TiO_2_ because it does not have d orbit, but the intrinsic defect states like oxygen vacancy (*V_o_*) could be suppressed by Mg doping (Figure 7a). Moreover, the 2p orbit of Mg dopant apart from the level of conduction and valence band of TiO_2_ could change the level of defect states by hybridizing the oxygen vacancy in titania (Figure 7b). Besides, as a narrow band-gap semiconductor, Ta_3_N_5_ also has numerous photo-generated carrier recombination centers, which seriously impairs its photocatalytic activity. Recently, Xiao et al. [62] prepared Mg-doped Ta_3_N_5_ hollow spheres using carbon spheres templating. Mg ions doped into Ta_3_N_5_ could not only increase the charge mobility but also tune the electronic structure to promote the charge transportation. From the result of Mott–Schottky measurement (Figure 7c), Mg-Ta_3_N_5_ had a higher electron density. Theoretically, Mg ions acted as an electron acceptor in Ta_3_N_5_ photocatalysts. Mg doping was relatively efficient strategy, its photocatalytic hydrogen production is about 7.6-times that of prinstine Ta_3_N_5_ (Figure 7d).

Researchers have successfully achieved the doping of different elements in various materials by using carbon sphere template method. According to the results, the distribution of doping elements is uniform and there is no phase separation. For some doping systems that cannot be achieved by other preparation methods, we can try to achieve it by the non-selective adsorption of carbon sphere templates. This also provides ideas for other systems such as single atomic catalysis. Different kinds of salts can be used to make the expected elements adsorb and anchor on the surface of the template, and metal particles with good dispersion may be obtained through subsequent treatment.

### 3.2. Solid Solution

**Figure 8:**
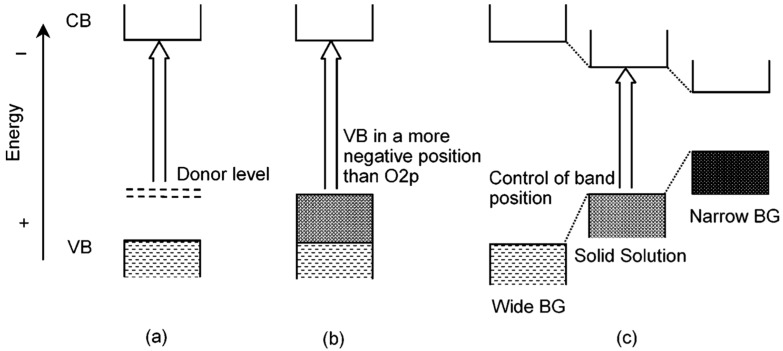
Band engineering through doping and forming solid-solution. (**a**,**b**) Formation of a new VBM by doping a foreign element to create a donor level, and (**c**) forming solid solution to obtain a controllable bandgap. Reproduced with permission from [64]. Copyright American Chemical Society, 2004.

**Figure 9:**
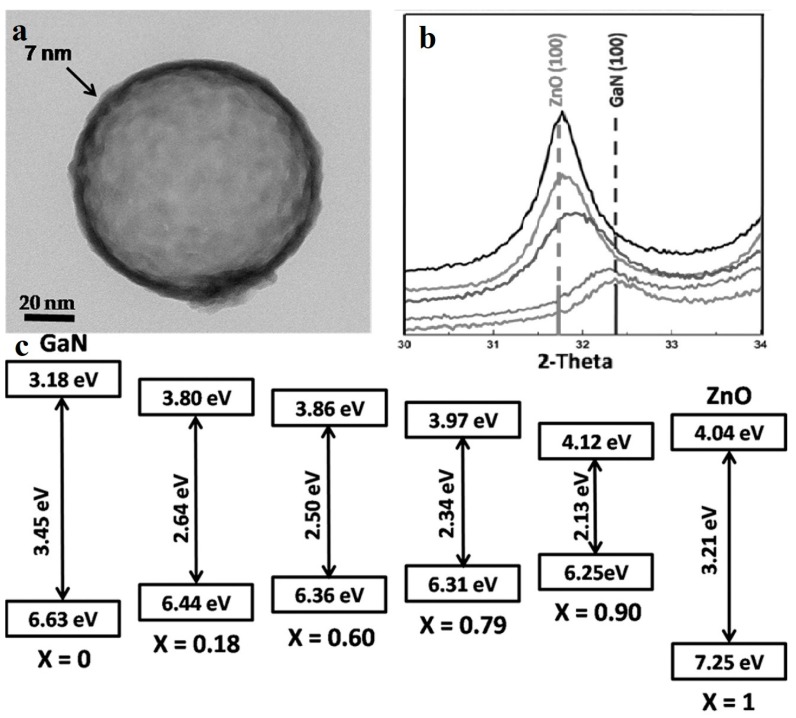
(**a**) TEM image shows an extremely thin shell (about 7 nm) of (Ga_0.82_Zn_0.18_)(N_0.82_O_0.18_) hollow spheres. (**b**) The (100) peak shift between GaN (100) and ZnO (100) with different *x*. (**c**) The band structure of the (Ga_1-*x*_Zn*_x_*)(N_1-*x*_O*_x_*) nanostructures with different x. Reproduced with permission from [69]. Copyright WILEY, 2015.

The bandgap engineering of a semiconductor is very important for its application in various aspects. It not only determines the optical absorption edge and emission wavelength of the material, but also closely related to the electrical conductivity [63]. However, it is difficult to find some semiconductors in nature whose bandgaps fit the requirements of various applications precisely, and that makes bandgap engineering an indispensable study. In order to tailor the bandgap and optoelectronic property of semiconductors, scientists have come up with two main solutions, including doping and forming solid solutions (Figure 8) [64]. The doping method has been described above. Forming solid solutions is to synthesize two or more different semiconductors (with similar crystal or electronic structure) into one material, and the bandgap-controllable solid solutions can be obtained by fine-tuning the content of different components. When applied to photocatalysis, the band gap is expected to be quite narrow while satisfying the potential of the catalytic reaction. Similar to the strategy of doping in previous section, carbon sphere template’s uniform and non-selective adsorption of metal ions also enables the synthesis of solid solution hollow nanostructure. By precisely controlling the precursor concentration, a broad solid solubility can be achieved. 

(Ga_1-*x*_Zn*_x_*) (N_1-*x*_O*_x_*) is formed by GaN and ZnO. Unlike a solid solution synthesized by a wide and a narrow band semiconductor, GaN and ZnO both have wide bandgaps, while their solid solution has a narrow bandgap about 2.58 eV when the content of ZnO is 13.3% [65]. It is calculated that the VBM (valence band maximum) is composed of Zn 3d orbitals and *N* 2p orbitals, and the repulsion of electrons of Zn 3d and *N* 2p result in the narrowing of the band gap [66], which makes the material more suitable for photocatalysis than sole GaN and ZnO. The water-splitting quantum yield of the bulk (Ga_1-*x*_Zn*_x_*)(N_1-*x*_O*_x_*) is limited by carrier migration, trapping and recombination. Maeda et al. [67] enhanced the quantum efficiency to 5.9% at 420–440 nm by surface modification, optimizing annealing temperature and composition *x* of bulk (Ga_1−*x*_Zn*_x_*)(N_1−*x*_O*_x_*). Many scientists have improved the overall conversion efficiency of the system by preparing nanomorphological materials. Reinert et al. [68] obtained nanorods arrays with diameter of about 100 nm. Their Ga_2_O_3_(ZnO)_16_ precursor was heat-treated and cooled under NH_3_ flow. By reducing the synthesis temperature to 850 °C, the volatilization of Zn was also greatly reduced, thus increasing the *x* value from <0.42 to 0.55 [65], and reducing the band gap to 2.53 eV. Li et al. [69] synthesized (Ga_1−*x*_Zn*_x_*)(N_1−*x*_O*_x_*) nano-hollow spheres by ion adsorption and templating. They used carbon spheres as templates, DMF as solvent, supplemented with the subsequent nitridation, and obtained various composition of (Ga_1−*x*_Zn*_x_*)(N_1−*x*_O*_x_*) by adjusting the ratio of the Zn source to the Ga source, tuning the composition of ZnO to the full range from 0.18 to 0.95. The magnified TEM image (Figure 9a) shows the nano-hollow-spheres, whose diameter are approximately 100–120 nm and the shell of the sphere is about 7 nm thick. Figure 9c indicates that using this new synth etic method, the narrowing of the bandgap is related to the mutual approachment of both VBM and CBM (conduction band minimum). The nitridation temperature was only 600 °C attributing to the the nanoscale hollow structures, much lower than that of bulk form which is approximately 900 °C [70], thus it reduces the volatilization of Zn. This nanoscale structure also decreases the carrier diffusion distances and increases the number of reaction sites, enhancing the quantum efficiency to 17.3% (400 nm), which was about 3 times higher than bulk (Ga_0.82_Zn_0.18_)(N_0.82_O_0.18_) [67].

A similar situation exists in some other solid solution systems. Zn*_x_*Cd_1-*x*_S is a solid solution composed of CdS and ZnS, which not only has tunable band gap, but also has better photocorrosion resistance and higher photocatalytic activity than pure CdS [71,72]. Gholipour et al. [73] synthesized Zn*_x_*Cd_1-*x*_S hollow spheres by a solvothermal method, using glycerin to obtain carbon microspheres [74], which contains large amount of OH radicals that can adsorb Zn^2+^ and Cd^2+^. After centrifugated, calcinated and calcinated in H_2_S/Ar flow, the Zn*_x_*Cd_1-*x*_S hollow spheres can finally be obtained. The value of x can be tuned by adjusting the ratio of zinc source to cadmium source.

**Figure 10:**
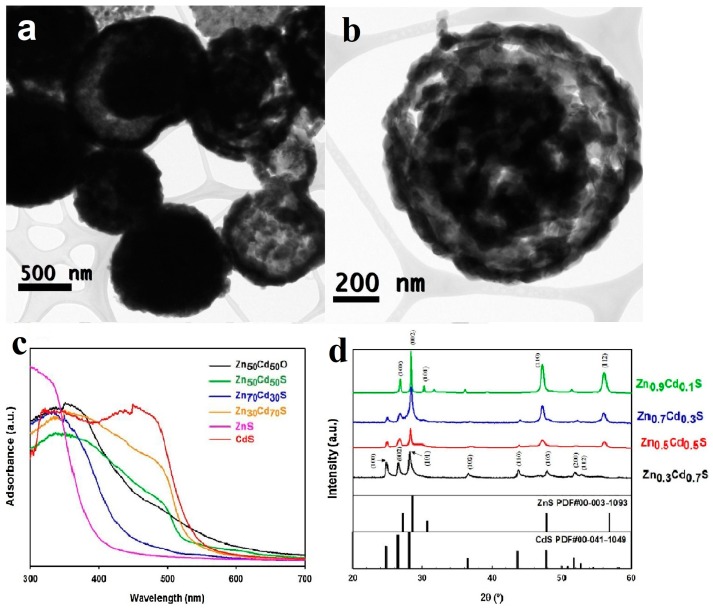
(**a**,**b**) TEM images of Zn*_x_*Cd_1-*x*_S hollow sphere. (**c**) UV–vis spectra of: Zn_0.5_Cd_0.5_O, Zn_0.5_Cd_0.5_S, Zn_0.7_Cd_0.3_S, Zn_0.3_Cd_0.7_S, ZnS, and CdS (**d**) XRD patterns of solid solutions of Zn*_x_*Cd_1-*x*_S after H_2_S treatment. Reproduced with permission from [73]. Copyright Elsevier, 2018.

The nanostructure of Zn*_x_*Cd_1-*x*_S is hollow sphere with an average diameter of 500 nm (Figure 10a,b). With the increase of Cd content, the absorption edge moves towards longer wavelength (Figure 10c), indicating the decrease of bandgap. At the same time, the XRD (X-ray diffraction) peak of the samples gradually shifted to lower angle (Figure 10d), accounting for the transformation from hexagonal ZnS to hexagonal wurtzite CdS. The hydrogen evolution they received is as high as 12 mmol h^−1^ g^−1^ under solar irradiation using MoS_2_ as cocatalyst. The calculated quantum efficiencies (QE) reached to 46.6% at 400 nm, which was among the highest QE in this system.

So far, there are few solid solution systems realized by carbon sphere template method. By adjusting the precursor and subsequent treatment, this method may be able to achieve more solid solution hollow nanostructures.

### 3.3. Heterostructure

It is hardly for individual semiconductor materials to satisfy fundamental requirements of highly efficient photocatalysts, such as suitable bandgap for effective sun light absorption, proper charge separation, high carrier mobility and fast kinetics for surface reaction [75,76]. Introducing heterojunction comes to be a potential strategy to meet these critical requirements simultaneously by combining the advantages of two or more semiconductors. A conventional heterojunction is established by coupling two semiconductors having suitable structures of energy band directly. Heterostructured hollow photocatalysts have received growing research attention by cause of their unusual advantages such as ultrahigh sunlight absorption, extremely large surface area and short carrier transfer distance in recent year [76,77]. So far, heterostructured hollow photocatalysts have demonstrated great application potential in varied photocatalytic fields [49,78]. Due to the good adsorption capacity of carbon sphere template, we are able to obtain hollow nano heterostructures by multiple adsorption and different annealing conditions.

Semiconductor-based photocatalysts applied in water splitting are identified as the most economic and environmentally friendly material systems for hydrogen production [79]. In recent years, a great deal of previous research spotlighted on water splitting based on advanced heterostructured hollow photocatalysts.

**Figure 11:**
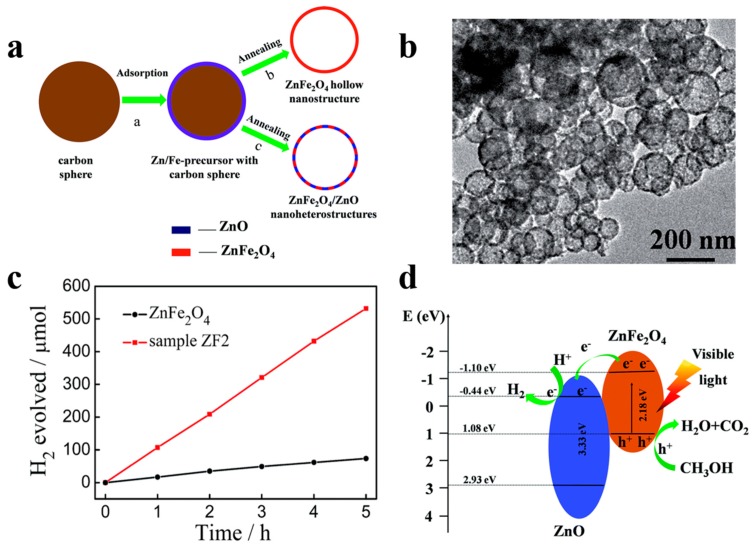
(**a**) Schematic diagram of preparation process of ZnFe_2_O_4_ nanostructures and ZnFe_2_O_4_/ZnO nanoheterostructures. (**b**) TEM images of ZnFe_2_O_4_/ZnO spheres. (**c**) H_2_ production over time by 50 mg photocatalysts of bare ZnFe_2_O_4_ and ZnFe_2_O_4_/ZnO (ZF2) sample driven by visible light (>420 nm). (**d**) Schematic illustration of ZnFe_2_O_4_/ZnO hetrtojunction energy band position and predicted reaction mechanism under irradiation. Reproduced with permission from [80]. Copyright Royal Society of Chemistry, 2015.

**Figure 12:**
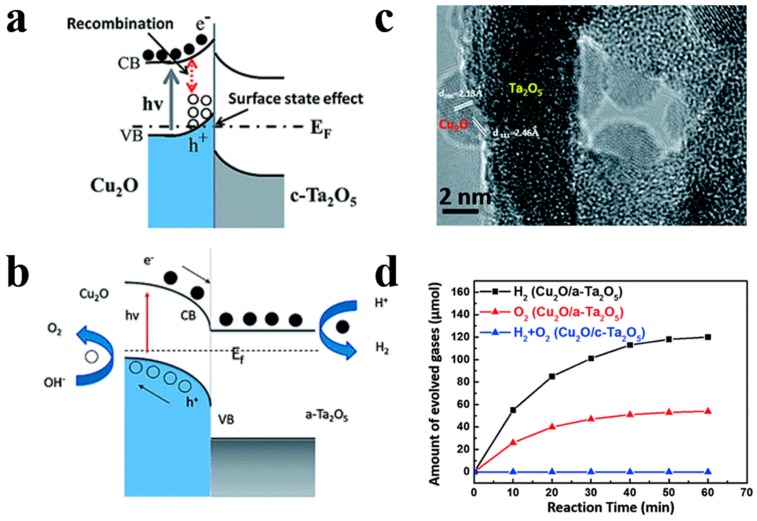
(**a**) Band bending diagram of Cu_2_O/crystalline Ta_2_O_5_. (**b**) Band bending diagram of Cu_2_O/amorphous Ta_2_O_5_ nanoheterojunctions. (**c**) HRTEM image of Cu_2_O/amorphous Ta_2_O_5_heterostructures. (**d**) Amount of evolved gases under full light for different samples. Reproduced with permission from [82]. Copyright Royal Society of Chemistry, 2017.

For instance, Song et al. [80] demonstrated the superior photocatalytic H_2_ evolution activity of ZnFe_2_O_4_/ZnO hollow nanoheterostuctures by a facile hard-templating approach. The nanoheterostuctures of ZnFe_2_O_4_/ZnO, which are about 15 nm in size, are homogeneously distributed in the hollow sphere shell. The champion sample without the help of co-catalysts exhibits an outstanding H_2_ evolution rate of 2.15 mmol h^−1^ g^−1^ when irradiated by visible light. The corresponding quantum efficiency reaching 1.61%, which is 7.6 times higher than that of bare hollow ZnFe_2_O_4_ photocatalysts. ZnO forms a type-II heterojunction with ZnFe_2_O_4_ in the sphere shell (Figure 11) which confirmed by the combination investigation of XPS (X-ray photoelectron spectroscopy) technic as well as UV–vis diffuse reflectance spectroscopy. The photogenerated electrons and holes will be separated into the conduction band of ZnO and valence band of ZnFe_2_O_4_ by the built-in electric field, respectively, thereby minimizing the recombination and boosting the photocatalytic H_2_ generation performance of samples. Meanwhile, Li et al. [81] have reported a high-crystallinity g-C_3_N_4_/TiO_2_ hollowsphere nanoheterojunction photocatalyst with large surface area through a carbon sphere templating method. By carefully tuning the ratio of precursors, the optimum g-C_3_N_4_/TiO_2_ photocatalyst obtain a 5.5-fold enhancement in visible-light-driven hydrogen generation rate, reaching almost 470 μmol g^−1^ h^−1^. The improvement in photocatalytic performance primarily owes to the establishment of heterojunctions which accelerating carrier’s separation efficiently confirmed by PL spectrum and electrochemical independence spectroscopy. While conventional heterojunctions are generally in form of crystalline/crystalline contact, Lou et al. [82] have investigated a novel type of crystalline/amorphous heterostructure. A crystalline Cu_2_O/amorphous Ta_2_O_5_ heterostructure fabricated by a two-step ion absorption approach based on carbon spheres template. Such heterostructures were found to possess the capacity for splitting water into H_2_ and O_2_ driven by visible light without using sacrificial agent. Theoretical calculation shows the existence of hole trap states in the crystalline/crystalline interface cause by lattice and electron mismatch, which does not exist at the crystal/amorphous interface (Figure 12). Moreover, the type-II heterojunction of crystalline Cu_2_O/amorphous Ta_2_O_5_ greatly promotes the separation of photo-induced charges to perform effective overall water splitting.

In addition, other research groups also have reported hollow heterojunction photocatalysts with high performance. Waqas et al. [83] prepared hollow sphere TiO_2_/Fe_2_TiO_5_ heterojunction photocatalyst through a simple two-step templating method for high efficient solar water oxidation. The oxygen evolution reaction rate of the champion sample with 35% Fe reached 375 μmol h^−1^ g^−1^ benefited from the desirable energy band alignment of TiO_2_/Fe_2_TiO_5_ heterojunction, which improved carrier separation in these photocatalysts. In another work, heterostructured ZnFe_2_O_4_/ZnSe hollow sphere photocatalysts which were synthesized through two-step carbon templating approach used in visible-light-driven water splitting [84]. Heterostructured ZnSe photocatalysts with suitable shell thickness generated a large amount of H_2_ of 16.9 mmol g^−1^ in 6 h, owing to the effective charge separation and accelerated carrier transfer.

Hollow nanoheterostructured photocatalysts possess extremely large surface area, high catalytic activity and carrier separation efficiency, thus being suitable for application of pollutant degradation [49,75,77,85].

**Figure 13:**
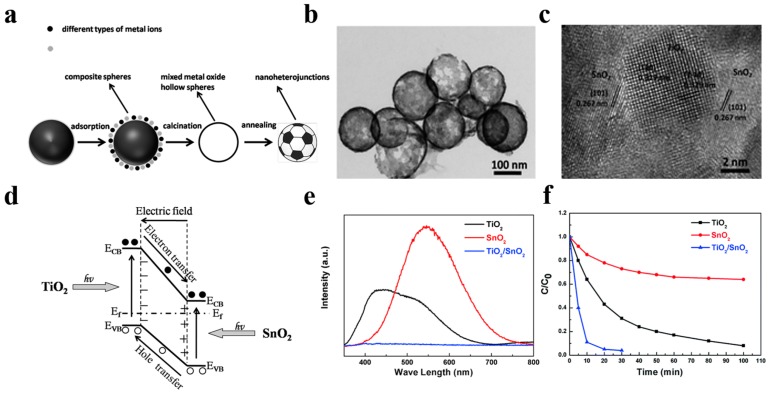
(**a**) Schematic illustration of the formation path of nanoheterostructured photocatalysts. (**b**) TEM image of the TiO_2_/SnO_2_ hybrid nanostructure. (**c**) HRTEM images of the TiO_2_/SnO_2_ nanoheterojunctions. (**d**) Schematic illustration of band structure of TiO_2_/SnO_2_ heterojuction. (**e**) The PL spectra of three different hollow spheres, red line for SnO_2_, black line for TiO_2_, and blue line for TiO_2_/SnO_2_. (**f**) The photocatalytic activity measurement of different structures towards RhB under full light irradiation. Reproduced with permission from [86]. Copyright Royal Society of Chemistry, 2014.

In 2014, Li et al. [86] have pioneered a simple and universal method for fabricating a hybrid hollow nanoheterostructured photocatalyst with ultrathin shell. Taking the TiO_2_/SnO_2_ hollow sphere photocatalyst as a typical example, they mixed 0.5 g of carbon spheres and a proper amount of C_16_H_36_O_4_Ti and SnCl_2_·2H_2_O together and stirred for 24 h, followed by rinsing and drying in an oven at 80 °C for 6 h. The TiO_2_/SnO_2_ hybrid nanostructured hollow spheres finally obtained by calcination have 3–10 nm small grain size and ultrahigh surface area of 300 m^2^ g^−1^. Compared to single TiO_2_ and SnO_2_ hollow nanostructured photocatalysts, the TiO_2_/SnO_2_ hybrid hollow nanostructured photocatalyst shows a superior RhB dye photodegradation activity, which is profited from the built-in electric field induced by TiO_2_/SnO_2_ heterojunction as well as the short carrier diffusion length in TiO_2_/SnO_2_ nanocrystal (Figure 13). Adopting a similar strategy, Lou et al. [87] have also successfully prepared a hollow Fe_2_TiO_5_/TiO_2_ nanoheterostructure to enhance the photocatalytic performance of TiO_2_-based photocatalysts driven by visible light. The favorable energy band alignment of Fe_2_TiO_5_/TiO_2_ heterojunction suppressed the carrier recombination significantly. As a result, the optimum nanoheterostructured photocatalyst exhibited a 3-times higher visible-light photocatalytic-degradation activity towards RhB compared to that of commercial P25 photocatalyst.

**Figure 14:**
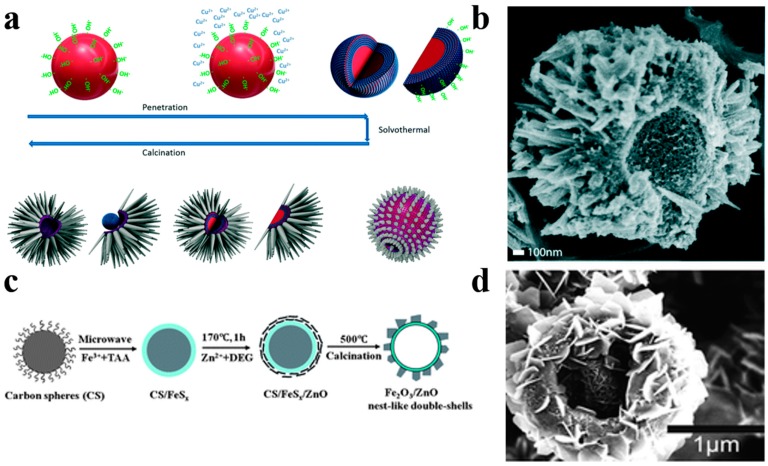
(**a**) Schematic diagram of the fabrication process of hollow hierarchical CuO/TiO_2_ structure. (**b**) SEM image of the CuO/TiO_2_ spheres. Reproduced with permission from [85]. Copyright 2017, RSC. (**c**) Schematic diagram of the preparation process of nest-like *γ*-Fe_2_O_3_/ZnO hollow nanostructures. (**d**) SEM images of *g*-Fe_2_O_3_/ZnO nest-like hollow nanostructures. Reproduced with permission from [88]. Copyright Royal Society of Chemistry, 2012.

Diversification of structural morphology makes hollow sphere photocatalysts more attractive. Ang et al. [85] have reported multi-dimensional hierarchical hollow heterojunctioned CuO/TiO_2_ spheres with TiO_2_ thorns grown on the surface of CuO hollow spheres by a carbon spheres assisted template method (Figure 14a,b). The hierarchical TiO_2_ thorns acted as water channels generating a mass of reactive oxygen species for pollutant degradation. The multi-dimensional CuO/TiO_2_ spheres show an excellent photodegradation activity in comparison to P25 thanks to the effective carrier separation and migration origin from the establishment of CuO/TiO_2_ heterojunction. Liu et al. [88] have successfully used a microwave-assistant carbon sphere templating method to synthesize hollow nest-like *γ*-Fe_2_O_3_/ZnO photocatalysts, which ZnO nanosheets were loaded on the shell of g-Fe_2_O_3_ hollow spheres (Figure 14c,d). The large surface area and effective restraint of carrier recombination of g-Fe_2_O_3_/ZnO result in an enhanced photocatalytic activity compared with bare ZnO photocatalysts.

Other type-II hollow heterostructured photocatalysts derived from carbon sphere template such as CeO_2_/TiO_2_ [89], WO_3_/TiO_2_ [90], ZnO/CuO [91], ZnO/SnO_2_ [92], and ZnO/ZnS [93] also showed enhanced photocatalytic pollutant degradation performance by the promotion of heterojunction.

Nanoheterostructure is one of the most facilely realized structures by carbon sphere template method. One-step adsorption by the carbon sphere template can realize heterostructure on the same spherical shell, and multi-step adsorption can realize hollow spherical nanostructures with core–shell distribution or multilayer structure. Notably, as powder photocatalysts, recycling is a very important issue. Generally, we build a heterostructure with magnetic materials to achieve magnetic recycling. However, magnetic materials generally have low photocatalytic activity and light blocking, which will reduce the efficiency of light utilization. By carbon sphere template method, the magnetic materials may be confined in the hollow nanospheres of photocatalyst by the method of multi-step adsorption, so as to realize the light utilization efficiency and recyclability.

### 3.4. Surface Modification

For photocatalysis, the more electron–hole pairs are photogenerated and come into play, the higher the efficiency will be. However, the recombination is proven to harmfully impede the process of water-splitting or degradation resulting in a low efficiency [27,37,82]. Hollow structures can shorten the transport distance of carries from bulk to their proper catalytic sites on the surface, which suppress bulk recombination tremendously [37,77]. However, a majority of surface defects remain there and behave as another kind of recombination centers [94]. Meanwhile the extra electronic and optical states may have an influence on electronic structure or band structure [95]. Surface modification is the most effective measure to suppress surface recombination and increase active sites. Carbon sphere templates can absorb precious metals uniformly in solution, and also can be used as good skeleton support in subsequent treatment. The carbon sphere template derived ultra-thin hollow nanostructure also provides favorable conditions for the subsequent surface modification. Furthermore, combining surface modification and hollow structure will endow photocatalysts with both higher activity and better stability. 

**Figure 15:**
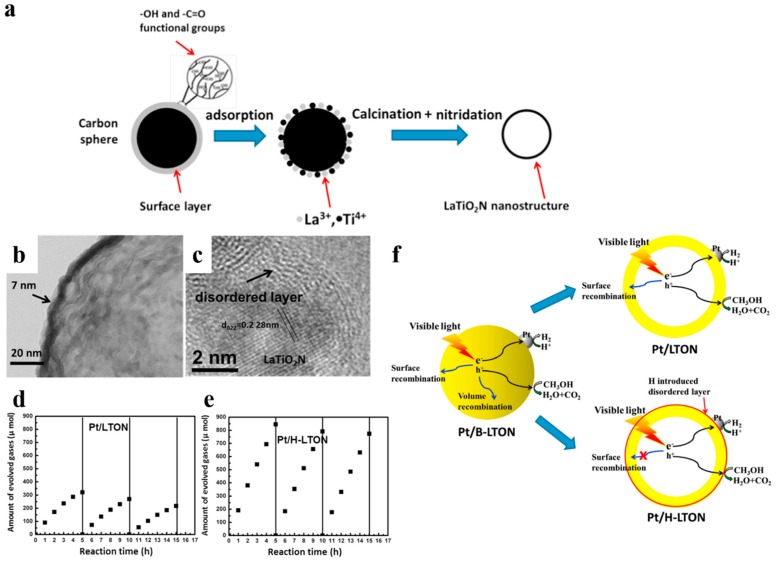
(**a**) The simple illustration of the synthesizing process of LaTiO_2_N nanostructures. (**b**) TEM image of LaTiO_2_N hollow nanostructures. (**c**) HRTEM image of the H_2_ treated LaTiO_2_N. (**d**,**e**) Hydrogen generation versus time on Pt/LTON and Pt/H-LTON irradiated by visible light (*λ* ≥ 420 nm) respectively. (**f**) Possible hydrogen generation principles proposed of the Pt/B-LTON, Pt/LTON and Pt/H-LTON respectively. Reproduced with permission from [95]. Copyright Elsevier, 2015.

The introduction of disorder at the surface of photocatalysts is a common method of surface modification. It has been revealed that lattice disorder in semiconductors could yield band tail states. It would dominate the optical excitation and relaxtion, and provide intended trapping sites for photo-generated carriers to avoid rapid recombination [96,97]. Chen et al. [96] prepared TiO_2_ nanocrystals with disordered surface layers via hydrogenation and found that it could help harvest infrared optical photons and contribute to organic molecules photo-oxidation and H_2_ evolution. Li et al. [95] reported a surface-reconstruced hollow LaTiO_2_N nanostructure with significantly improved photocatalytic activity (Figure 15). The ultrathin hollow LaTiO_2_N spheres were firstly synthesized by utilizing carbon spheres as hard templates. Then H_2_ treatment was carried not only to load the cocatalyst Pt but also to modify the surface of LaTiO_2_N spheres. Pt/H-LTON achieved a remarkably improved H_2_ evolution rate (up to 192 μmol h^−1^, compared to 91 μmol h^−1^ of Pt/LTON) and stability. Interestingly, the contrast sample with extra N_2_ annealing, which maintained the presence of Ti^3+^ ions but removed the disordered surface even exhibited worse photocatalytic performance than Pt/LTON. It indicated that the excellent photocatalytic capacity for water splitting was a consequence of the disordered surface rather than the induced Ti^3+^ ions. Introducing H atom can passivate surface defects, thus avoiding a majority of surface recombination of photo-induced carriers [94,95]. Therefore, surface disordering functions as a complementary strategy for improving photocatalytic activity combined with hollow structures.

**Figure 16:**
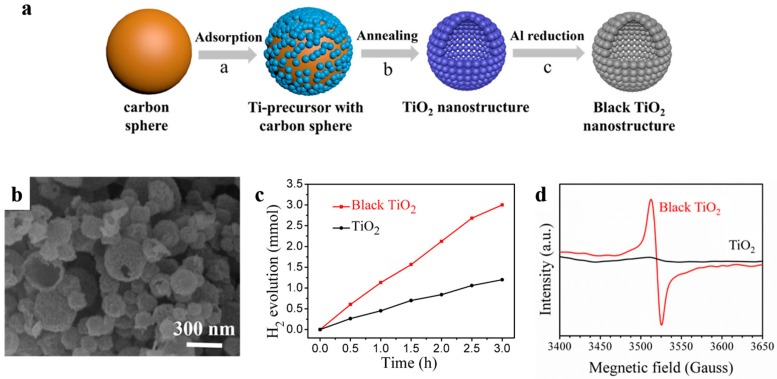
(**a**) The synthesis process of the as-prepared TiO_2_ nanostructures and black ones. (**b**) SEM image of black TiO_2_. (**c**) Time course of photocatalytic H_2_ evolution of TiO_2_ nanostructures. Reaction conditions: 20 mg photocatalysts, 1 wt % Pt loaded, under 300W Xe lamp irradiation. (**d**) EPR spectra of the as-prepared TiO_2_ hollow spheres and black ones. Reproduced with permission from [100]. Copyright American Chemical Society, 2017.

Oxygen vacancies are also proved to strongly affect photocatalytic activity of metal oxides-based photocatalysts [98]. Black titanium oxide is a typical example, in which electrons transition occurs between levels of valence band of the photocatalyst and the introduced oxygen vacancy states under visible radiation [98,99]. Recently, Song et al. [100] successfully prepared black titanium oxide hollow nanospheres with ultrathin shells for more effective solar-driven water splitting (Figure 16). The high quality TiO_2_ possessed a high specific surface area of 168.8 m^2^ g^−1^ and was obtained by a conventional carbon sphere templating method combined with subsequent Al reduction. This black TiO_2_ nanostructure exhibited a much higher photocatalytic H_2_ evolution rate (56.7 mmol h^−1^ g^−1^), which was well above that of the as-prepared one (22.9 mmol h^−1^ g^−1^) when using xenon lamp as irradiation source. It was found that Al reduction introduced masses of oxygen vacancies into TiO_2_ which can act as donors and accelerate the separation and transportation of the photogenerated electron–hole pairs.

**Figure 17:**
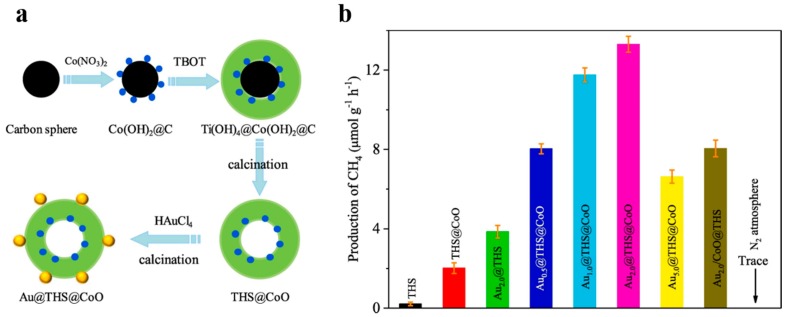
(**a**) Diagrams of preparation of Au_x_@THS@CoO. (**b**) Production rates of CH_4_ for THS, THS@CoO and Au_2.0_@THS@CoO. Reproduced with permission from [103]. Copyright Elsevier, 2019.

Besides, cocatalysts can help lower energy barriers, enhance photo-generated charge separation and provide reaction sites [101]. Different cocatalysts are designed to facilitate the oxidation or reduction process on the surface respectively [102]. Template method makes it easy to load related cocatalysts to the specified spots of photocatalysts. For example, Li et al. [102] designed Pt@TiO_2_@MnOx hollow spheres to improve the photo-oxidation of water and benzyl alcohol. Xiao et al. [62] reported that the Ta_3_N_5_ hollow spheres with Pt/CoOx dual cocatalysts exhibited better photocatalytic ability of water splitting compared with those only with Pt cocatalyst. Hollow TiO_2_ modified with spatially isolated Au and CoO dual cocatalysts for CO_2_ photoreduction was designed by Zhu et al. (Figure 17) [103]. CoO was formed on the inner surface of carbon spheres by ions absorption, and then Au was decorated on the external surface by Au ions reduction. The optimal formation rate of CH_4_ from CO_2_ reduction of Au_2.0_@THS@CoO was nearly 60 times as high as that of TiO_2_ hollow nanospheres (THS), while the activity of THS with Au and CoO nanoparticles randomly deposited was not so good. There is convincing evidence that Au and CoO are the suitable co-catalysts for photo-generated electrons and holes respectively so that carriers can be separated effectively on the inner or outer surfaces of the hollow nanostructure. Carbon templating is a convenient way of designing cocatalyst loading.

**Figure 18:**
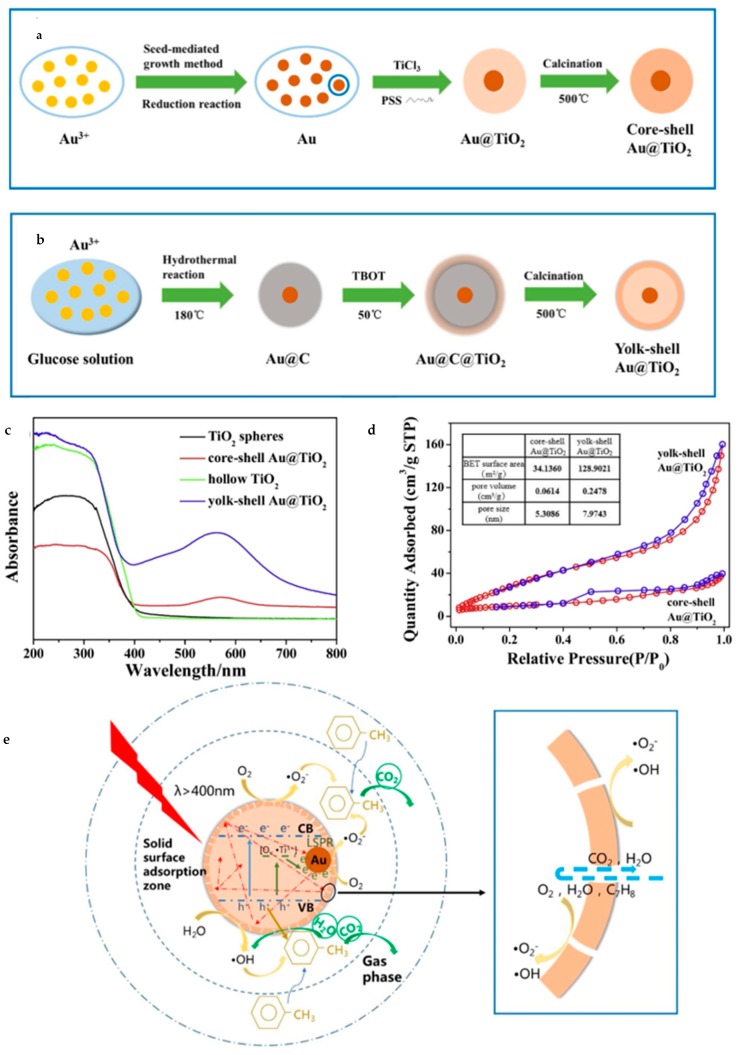
Schematic diagram of synthesis of (**a**) core–shell, (**b**) yolk–shell Au@TiO_2_. (**c**) UV–vis absorbance spectra for different kinds of TiO_2_ spheres. (**d**) The BET curves of yolk–shell and core–shell Au@TiO_2_ respectively. (**e**) The possible photocatalysis principle of Au@TiO_2_-YS. Reproduced with permission from [104]. Copyright Elsevier, 2019.

Furthermore, plasmonic metal nanocrystals have been intensively investigated recently on account of its unique optical and electronic properties. Gold (Au) and silver (Ag) can work as cocatalysts as well as light sensitizers in aspect of photocatalysis. Wang et al. [104] synthesized yolk–shell Au@TiO_2_ nanostructure by employing carbon spheres as templates (Figure 18). Core–shell Au@C was fabricated through hydrolysis and then it worked as a template to form yolk–shell Au@TiO_2_ (Au@TiO_2_-YS). The diameter of Au@TiO_2_-YS was about 350 nm with the Au core 70–90 nm or so. Evidently, this morphology brought a much larger BET (Brunauer–Emmett–Teller) surface area reaching 128.9 m^2^/g and a higher content of mesoporous particles than core–shell Au@TiO_2_. Owing to plasmonic effects, Au@TiO_2_-YS had broader light absorption extended to about 570 nm. It was found to be able to degrade the gaseous toluene more efficiently than the core–shell structure, almost 1.63 times higher. Obviously, the large surface area and abundant mesoporous channels induced by the yolk–shell structure make it easy to absorb more VOCs (Volatile Organic Compounds) and proceed the degradation. In addition, thin shells are also beneficial for photo-generated carriers to separate and migrate and unique cavities increase light reflection and refraction, too, thus enhancing the LSPR (Localized Surface Plasmon Resonance) effect of Au.

**Figure 19:**
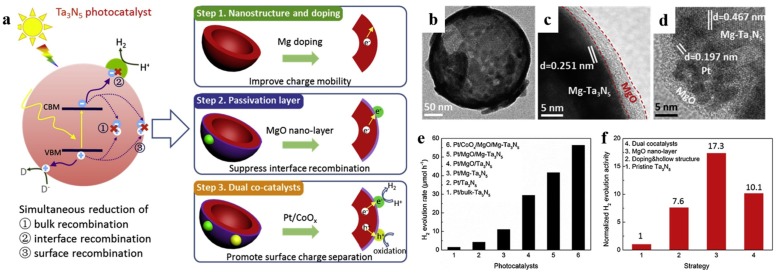
(**a**) Schematic diagram of how these three proposed approaches solve the recombination problems synergistically during the photocatalytic process of Ta_3_N_5_. Red, purple, green, and yellow objects represent Ta_3_N_5_, MgO, Pt, and CoO*_x_* respectively. TEM images of (**b**) Mg-Ta_3_N_5_, (**c**) MgO/Mg-Ta_3_N_5_ and (**d**) Pt/CoO*_x_*/MgO/Mg-Ta_3_N_5_ hollow spheres. (**e**) Hydrogen generation rates of Pt/Ta_3_N_5_, Pt/Mg-Ta_3_N_5_, Pt/MgO/Ta_3_N_5_, and Pt/CoO*_x_*/MgO/Mg-Ta_3_N_5_. (**f**) Comparison between the contributions of each approach to improve the photocatalytic ability of Pt/CoO*_x_*/MgO/Mg-Ta_3_N_5_ photocatalyst irradiated under simulated sunlight. Reproduced with permission from [62]. Copyright Elsevier, 2019.

In addition, passivation layers can also modify the surface of hollow structure. Xiao et al. [62] demonstrated that MgO nano-layer as passivation layer seemed to be an effective strategy for Mg-doped Ta_3_N_5_ hollow spheres (Figure 19). The Mg-doped Ta_2_O_5_ precursor fabricated with the aid of carbon spheres templating, and subsequently it was well wrapped in-situ by a thin layer of MgO even less than 5 nm. The particle size of Pt further dercreased from 2.04 nm to 1.33 nm along with the deposition of MgO. The increased ratio of hydrogen generation for MgO modification is 17.3 compared to the bulk Ta_3_N_5_, mainly due to the efficient charge transfer and passivation of surface defects. It should be noted that excessive MgO may adversely affect the charge transfer between Ta_3_N_5_ and cocatalysts instead since MgO is known as a insulator. Therefore, it is necessary to manage the thickness of the passivation layer by controlling the parameters of ion absorption.

Surface modification is not unique to the carbon sphere template method, but the adsorption characteristics of carbon sphere can well realize the delocalization of different co-catalysts. The carbon in the template can also acts as reducing agent. Under different post-treated atmosphere, it can play a role not only as a template. The hollow nanostructures with controllable shell thickness prepared by carbon sphere template method also provide convenience for many subsequent processing. In photocatalysis, the carrier migration distance is a very important factor. The thin shell prepared by carbon sphere template with subsequent surface treatment can greatly improve the efficiency of photocatalyst carrier utilization.

A summary of the recent carbon sphere template derived hollow nanostructure photocatalysts is presented in Table 1.

## 4. Carbon Sphere Template Derived Hollow Nanostructure for Gas Sensing

Hollow nanostructure also has widely applied in gas sensing since it possesses ultrahigh specific area which can provide rich active sites for gas adsorption and diffusion, further promoting gas-sensing performance. Moreover, the porous structures resulting in high surface permeability also allows more gas exposure and makes gas sensing easier [105,106,107]. Inspired by carbon sphere templating methods in photocatalysis, researchers have successfully implemented the modification strategies into gas sensing in view of carbon spheres templated hollow nanostructure with adjustable composition, thickness, surface, and interface [108,109,110].

**Figure 20:**
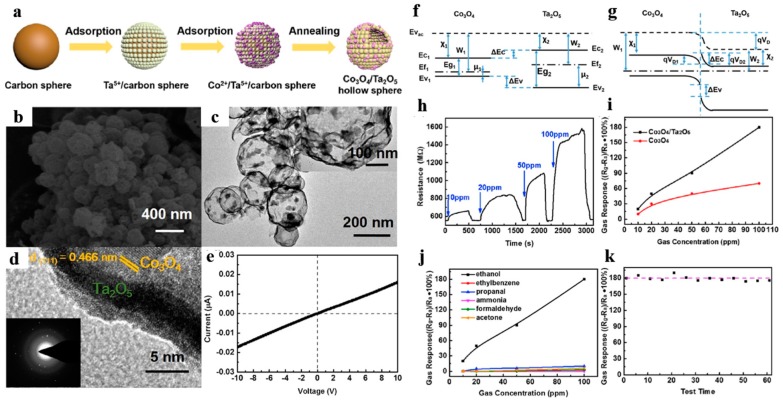
(**a**) Synthetic schematic of Co_3_O_4_/Ta_2_O_5_ heterostructure hollow nanospheres, (**b**) FESEM images, (**c**) TEM images, (**d**) HRTEM images. (**e**) I–V characteristics of the Co_3_O_4_/Ta_2_O_5_ sensor. Proposed energy band diagram: (**f**) before contact, (**g**) after contact. (**h**) Response and recovery curves of the Co_3_O_4_/Ta_2_O_5_ sensor at room temperature. (**i**) Sensitivity curves between the heterostructure and pure Co_3_O_4_ sensor with 10–100 ppm. (**j**) Selective test to several reducing gases with concentration of 10–100 ppm. (**k**) Long-term stability test during two months. Reproduced with permission from [111]. Copyright Elsevier, 2017.

For instance, Zhang et al. [111] firstly designed porous Co_3_O_4_/Ta_2_O_5_ heterojunction hollow nanospheres. They prepared ultrathin Ta_2_O_5_ hollow shell (shell thickness ~5 nm) attached with numerous Co_3_O_4_ nanoparticles by adsorbing Co ions onto Ta^5+^/C template spheres (Figure 20a–d). Figure 20e displayed that the gas sensor owned a good linear relationship of current vs. voltage between the sensing material and the electrode, ensuring that measured electrical signals were due to the gas-sensing performance rather than the contact resistance of the sensing material and the electrode. The gas sensor revealed splendid selectivity and reliable long stability to ethanol at room temperature (Figure 20h–k), which was attributed to two reasons: (1) the supporting effect of Ta_2_O_5_ hollow spheres hindered the agglomeration of Co_3_O_4_ particles and guaranteed larger BET area; (2) the formation of heterojunction resulted to widen the electronic depletion layer.

**Figure 21:**
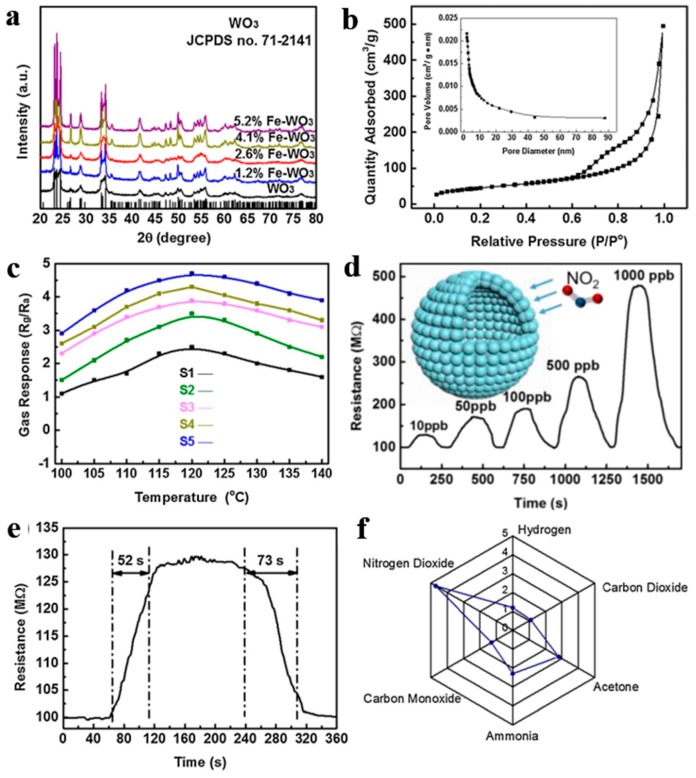
(**a**) XRD patterns. (**b**) N_2_ adsorption and desorption isotherms and pore diameter distribution curves. (**c**) Response of all samples with different Fe doping contents from 100 to 140 °C to 1000 ppb NO_2_. The gas-sensing performance of the representative S5: (**d**) dynamic sensing curve to different NO_2_ concentrations, (**e**) dynamic response curves to 10 ppb NO_2_ at 120 °C, and (**f**) selective test to 1 ppm NO_2_ versus several 10 ppm interfering gases. Reproduced with permission from [112]. Copyright Elsevier, 2018.

Furthermore, Zhang et al. [112] also synthesized WO_3_ mesoporous hollow nanospheres doped with Fe with a series of concentrations (0–5.2% by molar ratio) through a carbon colloidal nanosphere templating method, electrostatic adsorption of metal ions, as well as post-annealing treatment. This hollow structure showed tiny size and ultrahigh BET area. Gas sensor based on hollow nanostructured semiconductors finally realized outstanding performance applied for ppb-level NO_2_ detection at lower working temperature (100–140 °C) as exhibited in Figure 21. According to the results of XPS, the enhancement of NO_2_ detecting performance ascribed to rich oxygen vacancies and surface chemical states altered by Fe dopants. What is worth mentioning is that the excellent detection limitation can meet the needs of atmospheric monitoring and exclusive detecting at the same time.

**Figure 22:**
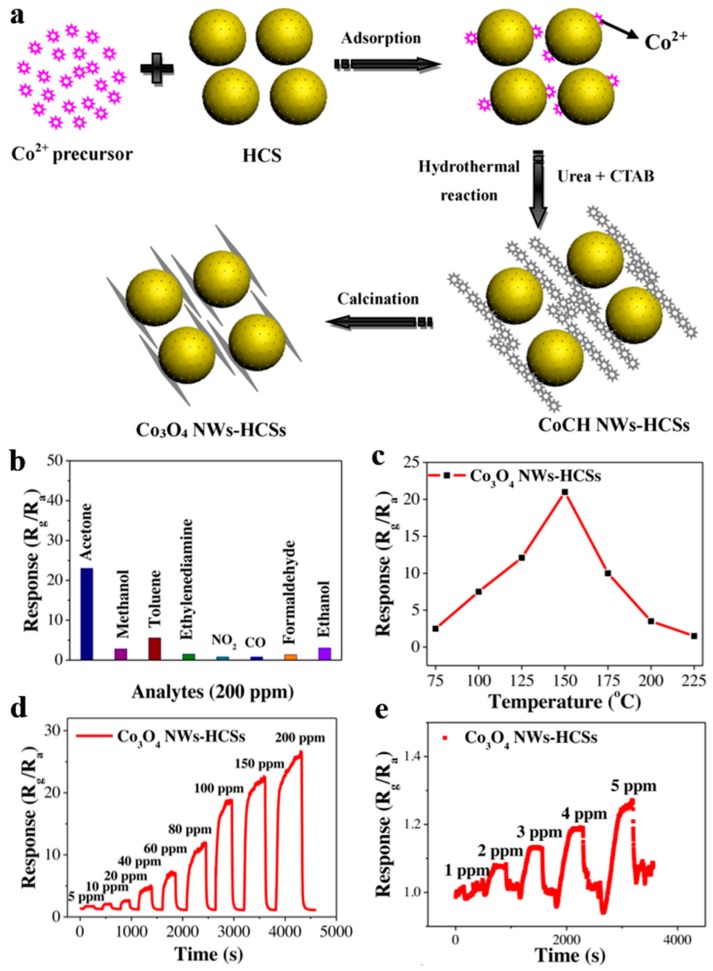
(**a**) Synthetic Schematic of Co_3_O_4_ NWs-HCSs. The gas-sensing performance of Co_3_O_4_ NWs-HCSs. (**b**) Sensing selectivity for several 200 ppm gases at 150 °C. (**c**) Sensing response versus operating temperature to 200 ppm acetone. (**d**,**e**) Dynamic sensing response to acetone within different concentration ranges at the operating temperature of 150 °C. Reproduced with permission from [113]. Copyright Elsevier, 2019.

Zhang et al. [113] studied hollow carbon spheres of which Co_3_O_4_ nanowires assembled on the surface (Co_3_O_4_ NWs-HCSs) by hydrothermal method together with subsequent heat treatment for acetone gas sensing (Figure 22a). Obviously, Co_3_O_4_ NWs-HCSs behaved talented selectivity for acetone, and whose response increased to 23 at 150 °C, even possessed high response at lower temperature of 75 °C, approaching room temperature.

**Figure 23:**
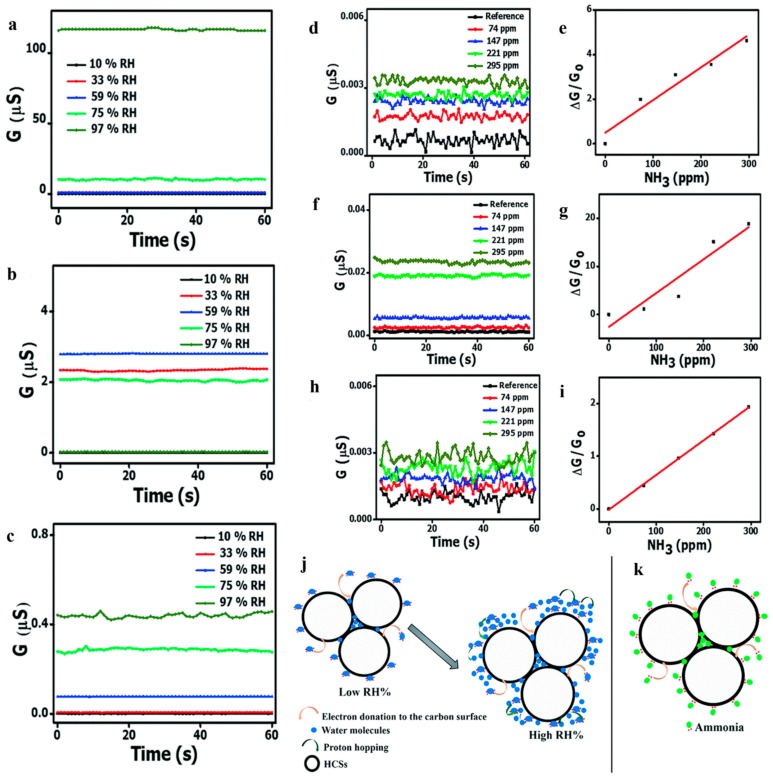
RH-dependent conductivity curves of (**a**) pure HCS, (**b**) HCS/PVP composite, and (**c**) annealed HCS based sensors at 40 °C. Ammonia concentration-dependent conductance and sensitivity of (**d**,**e**) pristine HCS, (**f**,**g**) HCS/PVP, and (**h**,**i**) annealed HCS based sensors at 10% RH at 40 °C. A schematic illustration of the sensing mechanism to NH_3_ of the HCSs at low RH (**j**) and high RH (**k**). Reproduced with permission from [114]. Copyright Royal Society of Chemistry, 2017.

Most researchers tend to study low temperature (even room temperature) and low concentration gas sensing taking the advantages of hollow structure, however they lacked research on humidity sensitivity. Notably, Mutuma et al. [114] reported that pristine hollow carbon spheres (HCSs), HCS/PVP (polyvinyl pyrrolidone) composite and annealed HCSs as ammonia sensors in damp conditions. The authors used a surfactant dispersion method to fabricate the HCSs and HCS/PVP. HCSs sensor should have responded in a high relative humidity (10–97% RH) owing to the presence of amorphous domains and oxygenated groups while high concentration of water molecules inhibited ammonia adsorption sites (Figure 23a). After adding PVP, HCSs still behaved obvious conductivity attenuation under high humidity resulting from polymer swelling and plasticization (Figure 23b). After subsequent annealing, the annealed HCSs obtained a great response to 74–295 ppm NH_3_ over a broad RH range (Figure 23c,h,i), decreasing the dependence on RH and raising surface area. This study provided insights into a step forward to modify the HCSs sensor in high humidity.

The above research presents several promising strategies to enhance gas-sensing performance through carbon sphere derived hollow nanostructure. Based on these studies, after further optimization on structure and process conditions, as well as material systems innovation, carbon sphere template derived hollow nanostructures would be promising in low temperature especially room temperature and ppb-level gas detection in high relative humidity environment, making it possible to apply in exhaled breath detecting in the future. A summary of the recent carbon sphere template derived hollow nanostructure gas sensors is presented in Table 2. Furthermore, hollow structures also have plenty of applications in the fields in energy storage [115,116].

## 5. Conclusion and Outlook

In summary, carbon sphere template derived hollow nanostructure has attracted considerable attention and achieved great progress in the fields of photocatalysis, gas sensing, etc. Among template methods, the use of highly hazardous, complex, inefficient, and costly fluoride-containing or strong acids and bases to etch the sacrificial templates limits their large-scale production and practical application. For these reasons, developing green, facile, and efficient strategies especially carbon sphere template derived hollow nanostructures with better photocatalysis and gas sensing performance is highly desirable and meaningful.

After tremendous efforts, the family of carbon sphere template derived hollow nanostructures have been broadened from monometal oxides to multimetal compounds. More importantly, doping, solid solution, heterostructure, and surface modification strategies have been successfully realized in carbon sphere template derived hollow nanostructures. These successful attempts are due to the unique characteristics of carbon sphere templates like the whole synthesis process is totally green and the surface radicals adsorb metal ions uniformly and non-selectively. This strategy is of great help to the three steps of photocatalytic process which enhancing light absorption, promoting carrier separation and benefiting surface redox reaction. This review summarized the recent development of carbon sphere template derived hollow nanostructures in photocatalysis. We summarize various synthetic routines of carbon spheres and carbon sphere template derived hollow nanostructures and highlight the mechanism for photocatalytic performance enhancement. The strategy has been also successfully applied in the field of gas sensing thus we present a summary of the recent progress in the development of carbon sphere template derived hollow nanostructures for gas sensing and related applications as well.

Despite significant progress in synthesis and effective property tuning of carbon sphere template derived hollow nanostructures, there are still some challenges and opportunities. For example, there are still many opportunities for research in the preparation of other types of fully adjustable solid solutions and tailoring bandgaps in most material systems. More systematic studies are needed to study the distribution of the minority atoms on the surface, such as whether the atomic dispersion can be achieved and applied to other reactions like photocatalytic CO_2_ reduction or nitrogen fixation. It is important to realize synthesis of configurationally disordered and entropy-stabilized mixed metal oxides, especially consist of earth-abundant elements, using this uniformly and non-selectively adsorption method. Any progress in the above urgent problems, will make great contributions to their corresponding application fields. We hope this review can give an overview of the research progress in carbon sphere template derived hollow nanostructures, so that researchers can have a more in-depth understanding of the process and application of carbon sphere templating method, and we have given a brief description on the outlook of future development, so as to promote photocatalysis and gas sensing to practicality and benefit the development of scientific researches in these fields.

## Figures and Tables

**Table 1:** Summary of carbon sphere template derived hollow nanostructure for photocatalysis.

Strategy	Material	Reaction	Efficiency	Reference
Doping	WO_3_: Fe	RhB degradation	*k*_RhB_ = 0.02 min^−1^	[52]
	ZnO: Na	H_2_ evolution	1380 μmol g^−1^ h^−1^ (AQY = 13.5% @ 350 nm)	[53]
	TiO_2_: Nd	*X*-3B degradation	*k_X_*_-3B_ = 0.0087 min^−1^	[54]
	TiO_2_: Ce	*X*-3B degradation	*k_X_*_-3B_ = 0.029 min^−1^	[55]
	TiO_2_: C	H_2_ evolution	35.6 μmol g^−1^ h^−1^	[56,57,58]
	TiO_2_: Br	RhB, MO, MB degradation	90% removal in 40 min	[59]
	LaTiO_2_N: Fe	O_2_ evolution	1.59 mmol h^−1^ (AQY = 55% @ 450 nm)	[60]
	TiO_2_: Mg	Overall water splitting	H_2_: 850μmol g^−1^ h^−1^ O_2_: 425μmol g^−1^ h^−1^ (AQY = 19.4% @ 350 nm)	[61]
	Ta_3_N_5_: Mg	H_2_ evolution	11 μmol h^−1^	[62]
Solid-solution	(Ga_1−x_Zn_x_)(N_1−x_O_x_)	Overall water splitting	H_2_: 254.2μmol h^−1^ O_2_: 127.1μmol h^−1^ (AQY = 14.3% @ 400 nm)	[69]
	Zn_x_Cd_1-x_S	H_2_ evolution	12 mmol h^−1^ g^−1^ (AQY = 46.6% @ 400 nm)	[73]
Heterostructure	ZnFe_2_O_4_/ZnO	H_2_ evolution	2.15 mmol h^−1^ g^−1^ (AQY = 1.61% @ 440 nm)	[80]
	g-C_3_N_4_/TiO_2_	H_2_ evolution	470 μmol g^−1^ h^−1^	[81]
	Cu_2_O/a-Ta_2_O_5_	Overall water splitting	H_2_: 202 μmol h^−1^ O_2_: 98 μmol h^−1^ (AQY = 3% @ 480 nm)	[82]
	TiO_2_/Fe_2_TiO_5_	O_2_ evolution	375 μmol g^−1^ h^−1^	[83]
	ZnFe_2_O_4_/ZnSe	H_2_ evolution	2.8 mmol g^−1^ h^−1^	[84]
	TiO_2_/SnO_2_	RhB degradation	*k*_RhB_ = 0.22 min^−1^	[86]
	Fe_2_TiO_5_/TiO_2_	RhB degradation	*k*_RhB_ = 0.09 min^−1^	[87]
	CuO/TiO_2_	AO7 degradation	*k*_AO7_ = 0.057 min^−1^	[85]
	γ-Fe_2_O_3_/ZnO	RhB, MO, MB degradation	90% RhB, MB removal in 50 min 80% MO removal in 80 min	[88]
	CeO_2_/TiO_2_	RhB degradation	75% removal in 180 min	[89]
	WO_3_/TiO_2_	MB degradation	78% removal in 80 min	[90]
	ZnO/CuO	MB degradation	98% removal in 60 min	[91]
	ZnO/SnO_2_	TC degradation	81% removal in 180 min	[92]
	ZnO/ZnS	RhB degradation	99% removal in 40 min	[93]
Surface modification	Surface-reconstruced LaTiO_2_N	H_2_ evolution	192 μmol h^−1^ (AQY = 3.4% @ 440 nm)	[95]
	Black TiO_2_	H_2_ evolution	56.7 mmol h^−1^ g^−1^ (AQY = 90.6% @ 365 nm)	[100]
	Pt@TiO_2_@MnO_x_	Benzyl alcohol oxidation	31.22 μmol in 14 h (AQY = 63.14% @ 254 nm)	[102]
	Pt/CoO_x_@MgO_2_@Ta_3_N_5_	H_2_ evolution	56.3 μmol h^−1^ (AQY = 0.31% @ 400 nm)	[62]
	Au_2.0_@TiO_2_@CoO	CO_2_ reduction to CH_4_	13.3μmolh^−1^g^−1^	[103]
	Yolk–shell Au@TiO_2_	Toluene degradation	57% removal in 180 min	[104]

**Table 2:** Summary of carbon sphere template derived hollow nanostructure for gas sensing.

Strategy	Material	Gas	Response	Reference
Doping	WO_3_: C	acetone	5.1 @ 0.9 ppm, 300 °C	[109]
	WO_3_: Fe	NO_2_	1.3 @ 10 ppb, 120 °C	[112]
Heterostructure	Pd@In_2_O_3_	ethanol	159 @ 5 ppm, 350 °C	[108]
	Co_3_O_4_/Ta_2_O_5_	ethanol	1.8 @ 100 ppm, 25 °C	[111]
	Co_3_O_4_@C	acetone	23 @ 200 ppm, 150 °C	[113]
Surface modification	Annealed C	ammonia	66 @ 74 ppm, 40 °C	[114]
